# Nutrient reallocation between stem and leaf drives grazed grassland degradation in inner Mongolia, China

**DOI:** 10.1186/s12870-022-03875-4

**Published:** 2022-10-29

**Authors:** Jiayue Liu, Shuaizhi Lu, Changcheng Liu, Dongjie Hou

**Affiliations:** 1 Ministry of Education Key Laboratory of Ecology and Resource Use of the Mongolian Plateau & Inner Mongolia Key Laboratory of Grassland Ecology, School of Ecology and Environment, Inner Mongolia University, 010021 Hohhot, China; 2 State Key Laboratory of Vegetation and Environment Change, Institute of Botany, Chinese Academy of Sciences, 100093 Beijing, China; 3 College of Grassland, Resource and Environment, Inner Mongolia Agricultural University, 010019 Hohhot, China

**Keywords:** Grazing, Macroelements, Microelements, Nutrient reallocation, Steppe

## Abstract

**Background:**

Decline in height and aboveground biomass of the plant community are critical indicators of grassland ecosystem degradation. Nutrient reallocation induced by grazing occurs among different organs, which balances the trade-off between growth and defense. However, it is not yet clear how nutrient reallocation strategies affect plant community structure and functions in grazed grasslands. A grazing experiment was conducted in a typical steppe in Inner Mongolia, China. We investigated plant community characteristics and measured plant functional traits of dominant species (*Leymus chinensis* and *Cleistogenes squarrosa*) at individual and population levels. Carbon (C), nitrogen (N), phosphorus (P), copper (Cu), iron (Fe), manganese (Mn), and zinc (Zn) concentrations of stem and leaf in the two species were also determined.

**Results:**

N, P, Cu, Fe, Mn, and Zn concentrations in leaves and stems of *L. chinensis* and *C. squarrosa* significantly increased with grazing intensity, and microelements (Cu, Fe, Mn, and Zn) were more sensitive to grazing. The nutrient slopes of macro- and microelements in leaves were significantly higher than those in stems under grazing, indicating that nutrient resources were preferentially allocated to leaves and enhanced the compensatory growth of leaves in the grazed grassland. With increasing grazing intensity, the aboveground biomass of stems and leaves in the two species significantly decreased, but leaf to stem ratio increased at the individual level, indicating that plants preferentially allocated biomass to leaves under grazing. The increase in leaf to stem ratio due to nutrient reallocation between the two organs significantly reduced height and aboveground biomass at population and community levels, driving grassland ecosystem degradation.

**Conclusion:**

Our study revealed the driving forces of community structure and function degradation in grazed grasslands from the perspective of nutrient resource allocation, and provided insights into plant adaptation strategies to grazing.

## Introduction

Grassland ecosystems are important terrestrial ecosystems and have irreplaceable economic value and ecological functions [1, 2]. Climate change and overgrazing induce severe degradation of grassland ecosystems, decreasing ecosystem service and functions [3]. Decrease in height and aboveground biomass, and replacement of dominant species are critical indicators of degraded grassland ecosystems [4, 5]. Foraging and trampling of livestock are the main driving forces of grassland degradation [6, 7]. These two livestock behaviors cause intense damage to plants. However, compared with roots, plant shoots (e.g., morphology and nutrient characteristics) have sensitive and rapid regulation between growth and defense in grazed grasslands [8, 9]. Some studies have shown that overgrazing significantly decreased some leaf and stem traits, including shorter plants, narrower leaves, shorter internodes, smaller clusters and shallower roots [10, 11]. Miniaturized plants with shortened leaves and stems increase the foraging difficulty for livestock, which protects plants against grazing. More importantly, the plant community composed of these miniaturized individuals has lower height and aboveground biomass. This phenomenon is widespread in degraded grassland ecosystems [12–14]. Therefore, revealing the mechanisms behind decrease in plant height and aboveground biomass is key to elucidating processes of structural and functional degradation in grazed grassland ecosystems. Previous studies have reported multiple underlying mechanisms of this ecological process in grazed grasslands. For example, some studies showed an increase in secondary metabolites (e.g., tannins, total phenols, and abscisic acid concentrations) induced the decrease in plant stem and leaf traits under grazing [15, 16].Other studies indicated that the changes in microenvironment drove these ecological processes [17]. Ecological stoichiometry reveals changes in plant community structure and function by nutrient balance among multiple elements [18–19], which can provide new insights to elucidate this ecological process.

Competition occurs among plant roots, stems, and leaves for the utilization of limited nutrient resources during life history, resulting in allometric growth among these organs [20]. Grazing significantly alters the nutrient cycling of soil-plant systems in grassland ecosystems and decreases nutrient acquisition by plants [21, 22]. Thus, plants enhance the nutrient limitation among different organs during life history in grazed grasslands [23]. Plants will optimize resource allocation among different organs (e.g., leaves, stems, and roots) to balance the trade-off between growth and defense or adapt to environmental changes [24]. For example, some studies have found that plants allocated more nutrient resources to roots (e.g., increasing root to shoot ratio) in grazed grasslands [16, 25]. In fenced grasslands, plants increased nutrient allocation to stems rather than leaves [26]. Thus, nutrient allocation strategies could reflect the characteristics of tradeoff strategies in response to environmental changes. Plant stems and leaves are important functional organs and sensitive to environmental changes [11, 27]. Plant stems and leaves suffering from environmental disturbance could enhance nutrient competition, leading to nutrient reallocation between stems and leaves [28]. The combined effect of nutrient reallocation between stems and leaves induced by nutrient reallocation transfers to other organs regulates nutrient resource allocation at multiple hierarchies [11]. Thus, studying nutrient resource reallocation between stems and leaves could provide new insights into the response and adaptation of plants to environmental changes.

Most studies have shown that plants rapidly reconstruct damaged tissue by increasing nutrient concentrations [29]. Leaves are the main photosynthetic organ, but stems play important roles in mechanical support and material transport. In grazed grasslands, foraging and trampling by livestock severely damage leaf structure and decrease leaf area, which directly reduces photosynthesis and carbohydrates, and further indirectly affects other physiological processes, such as water use efficiency and nutrient acquisition [16]. Thus, the nutrient resources that plants require become more limited with increasing grazing intensity. Under the nutrient deficit induced by grazing, plants regulate nutrient allocation strategies to form ecological adaptation strategies in grazed grasslands [28]. The change in nutrient concentrations of leaves or plant shoots can be used to evaluate nutrient adaptation strategies [29–31]. Due to the different functions of stems and leaves in plant physiology, the two organs compete strongly for nutrients. Previous grazing experiments have investigated the different responses of nutrient concentrations in stems and leaves. Additionally, some studies have found that plant shoot nutrients had no significant response to grazing intensity [32]. This result might be due to the inconsistent responses of leaf and stem nutrients to grazing. Compared with macroelements, microelements have received less attention, although they play essential roles in physiological metabolism [33, 34]. For example, iron (Fe) is an important component of enzymes or electron-transport receptors in photosynthesis or respiration, and zinc (Zn) has important functions in the synthesis of nucleic acid in cells [35]. Previous studies have reported that microelement concentrations in plant shoots significantly increased with grazing intensity [29]. However, there have been few studies of microelement reallocation between leaves and stems in grazed grasslands. The reallocation of macro- and microelements between stems and leaves might alter allometry between these two organs, which would affect plant functional traits and could even impact community structure and functions. Thus, elucidating nutrient reallocation in stems and leaves can reveal plants’ adaptation strategies to environmental change during grazing, and provide a new perspective on changes in community structure and function of grazed grasslands.

Grassland ecosystems in Inner Mongolia are typical of grassland ecosystems in China, and have important functions in ecological security in northern China [3]. They are also critical parts of the Eurasian steppe. Grazing is one of the main grassland uses in Inner Mongolia and has induced grassland degradation. In the present study, a grazing experiment was conducted in a typical steppe in Inner Mongolia, China. Our specific objectives were (1) to determine the relationship of nutrient reallocation between stems and leaves with grazing intensity; and (2) to identify how nutrient reallocation strategies between stems and leaves affect plant functional traits and community structure and function from multiple aspects. We hypothesized that plants preferentially allocate macro- and microelements to leaves rather than stems under grazing. Due to the relative decline in nutrient allocation to stems, plant height might decrease, which drives the decreases in height and aboveground biomass at population and community levels. These ecological processes comprehensively induce the degradation of community structure and function in grazed grasslands. Our study aimed to comprehensively reveal nutrient reallocation of multiple elements between stems and leaves in grazed grasslands and enhance knowledge of driving forces affecting community structure and function degradation in grazed grasslands from the perspective of nutrient resource allocation.

## Materials and methods

### Study site

This experiment was conducted at a grazing platform of the Institute of Grassland Research, Chinese Academy of Agricultural Sciences (44°40.66’N, 116°28.32′E, a.s.l. 1100 m), located in the typical steppe in Xilingol, Inner Mongolia, China. This region has a temperate continental climate, with a hot and rainy summer and a cold and arid winter. The annual mean temperature is 0.5 ~ 1.0℃, with averages of -19.0℃ in January (the coldest month) and 21.4℃ in July (the hottest month). Annual mean precipitation is about 280 mm, of which 80% occurs from May to September (the growing season). According to the Chinese soil classification, the soil type is chestnut soil, with maroon humus layer (organic matter content is less than < 4%) and calcium carbonate deposits (at a soil depth of 30 cm). Vegetation in this region is temperate typical steppe. *Leymus chinensis*, *Cleistogenes squarrosa* and *Stipa krylovii* are the dominant species, and *Artemisia frigida*, *Salsola collina*, and *Potentilla tanacetifolia* are associated species in the plant community. *L. chinensis*, and *C. squarrosa* have relatively higher importance values, and play key roles in regulating community structure and function. This site was freely grazed before 2007 and fenced from 2007 to 2013 prior to this experiment. The vegetation and soil have effectively and rapidly restored during this period. According to meteorological station during 2014 to 2018, the temperature and precipitation of this region were 0.8℃ and 262 mm, which were close to the long-term average precipitation and temperature.

### Experimental design

The grazing platform was established in the spring of 2014 with a complete random design. The experiment treatments included no grazing (CK, i.e. control treatment), moderate grazing (MG) and heavy grazing (HG) with three replicates of each. Each plot was 1.33 × 10^4^ m^2^ (115 m × 115 m). Grazing livestock were adult sheep with an average weight of 31.5 kg. Moderate grazing intensity was set following grazing intensity guidelines regulated by the local government. Heavy grazing intensity was twice that of moderate grazing intensity. Thus, 0, 8, and 16 sheep were grazed in the no grazing, moderate grazing, and heavy grazing, respectively. Following the local traditional grazing period, the grazing experiment started in the middle of June of each year and ended in the middle of September (continual grazing in the growing season) and there was no grazing disturbance from livestock at other times. According to the *Parameters for Degradation, sandification and salinization of rangelands* (GB 19,377 − 2003), a decrease in community height and aboveground biomass of 21%~50% indicates moderate degradation, while decreases greater than 50% indicate heavy degradation. In this grazing platform, the decreases of community height and aboveground biomass in moderate grazing plot were 47.9% and 23.4% of those in the control treatment, and 82.2% and 87.4% in heavy grazing plot.

### Sample collection and measurement

Plant community characteristics were investigated in the middle of August (the peak of aboveground biomass) in 2019. Three 1 m × 1 m quadrats were equidistantly set along a line transect (50 m) in each treatment plot. First, species composition was recorded and three individuals of each species were randomly selected to measure natural height. Then, the aboveground part of each species was harvested and placed in envelopes, dried at 65℃, and weighed.

According to plant functional group, *L. chinensis* and *C. squarrosa* are perennial rhizome grass and perennial bunchgrass, respectively [36]. Three 1 m×1 m quadrats were equidistantly set along another line transect (50 m) in each plot. In each quadrat, five healthy and mature *L. chinensis* and *C. squarrosa* individuals (aboveground parts) were harvested and placed in envelopes, dried at 65℃ to constant weight. Stems and leaves of each individual were separated carefully and weighed. Finally, the leaves and stems of individuals in the same treatment were mixed evenly to determine plant nutrients.

Plant samples were cut into small pieces (2 ~ 3 cm) with scissors and ground with a mill (MM400; Retsch). Carbon (C) and nitrogen (N) concentrations of stems and leaves were measured using a Vario EL (vario EL III CHNOS Elemental Analyzer, Elementar Analysensysteme GmbH). Phosphorus (P), magnesium (Mg), copper (Cu), Fe, Zn and manganese (Mn) concentrations of stems and leaves were measured using an ICP-OES (iCAP 6300 ICP-OES Spectrometer, Thermo Scientific) after sample digestion.

### Data analysis

The nutrient change rate indicates the range of variation in nutrient concentrations in stems and leaves under grazing and was calculated using the following equation [29]

Nutrient change rate = $\frac{Ci-Ck}{Ck}$ × 100%

where *C*_*i*_ indicates mean nutrient concentration of a species under a grazing treatment and *C*_*k*_ indicates mean nutrient concentration of this species under the control treatment.

Leaf to stem ratio indicates biomass allocation strategy between leaf and stem under a grazing treatment, and was calculated following the equation:

Leaf to stem ratio =$\frac{BL}{BS}$

where *B*_*L*_ and *B*_*S*_ are the dry aboveground biomass of leaf and stem of a species under a given treatment.

The normality of the data was tested and one-way analysis of variance was employed to test the differences in aboveground biomass of stems and leaves, nutrient concentrations of stems and leaves, and height and aboveground biomass at population and community levels among the no grazing, moderate grazing, and heavy grazing treatments. The multiple comparison LSD method was used. Linear regression of individual variables was used to analyze nutrient reallocation between stems and leaves with grazing intensity. The differences in nutrient slopes of the linear regression between stems and leaves were estimated using SMATR (Standardized Major Axis Tests & Routines) library (version 1) [37]. Structural equation modelling was used to reveal the critical ecological processes driving the changes in community structure and functions under grazing. The maximum likelihood method was used to construct the model and the accuracy of the model was evaluated by chi-square value, P value, R^2^ value and AIC value. Data is presented as mean ± 1SE and variance analysis was estimated at 0.05 level. Statistical analysis was conducted in IBM SPSS 24.0 and Amos 24.0 (SPSS Inc., Chicago, IL, USA).

## Results

### Macroelement and microelement characteristics of different organs under grazing

**Fig. 1:**
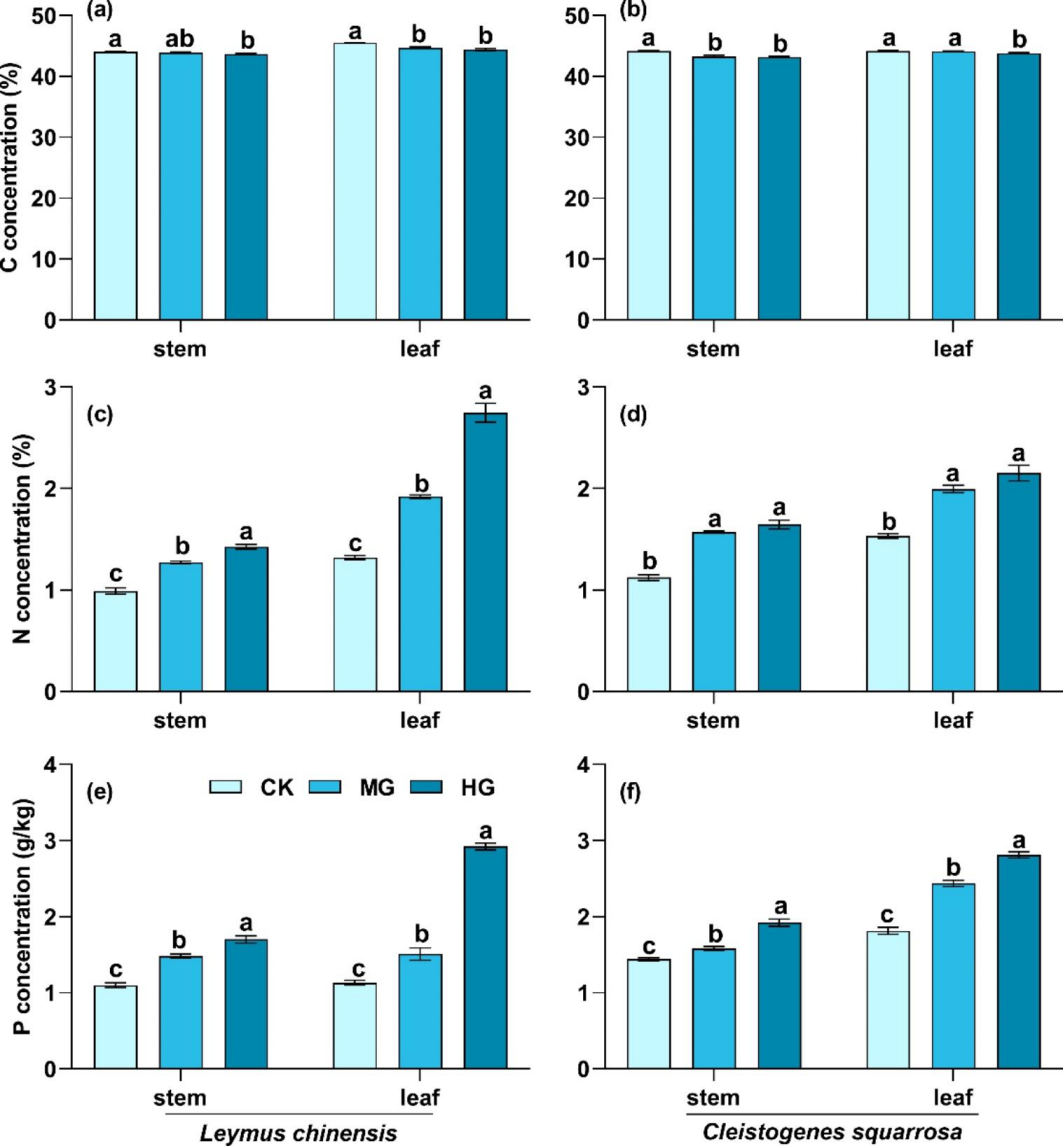
C, N, and P concentrations in stems and leaves of *L. chinensis* and *C. squarrosa*. (a), (c), and (e): *L. chinensis;* (b), (d), and (f): *C. squarrosa.* CK: control treatment; MG: moderate grazing; HG: heavy grazing. Different lowercase letters indicate significant differences in C, N, and P concentrations among the three grazing intensities at 0.05 level

Macroelements in the two species had divergent responses to grazing intensity, but the different organs had the same response to grazing. C concentration in stems and leaves of *L. chinensis* and *C. squarrosa* significantly decreased with grazing intensity, while N and P concentrations increased (Fig. 1; *P* < 0.05). For example, C concentration in stems and leaves of *L. chinensis* decreased by -0.9% and 2.5%, but N concentration increased by 44.1% and 108.1%, respectively.

**Fig. 2:**
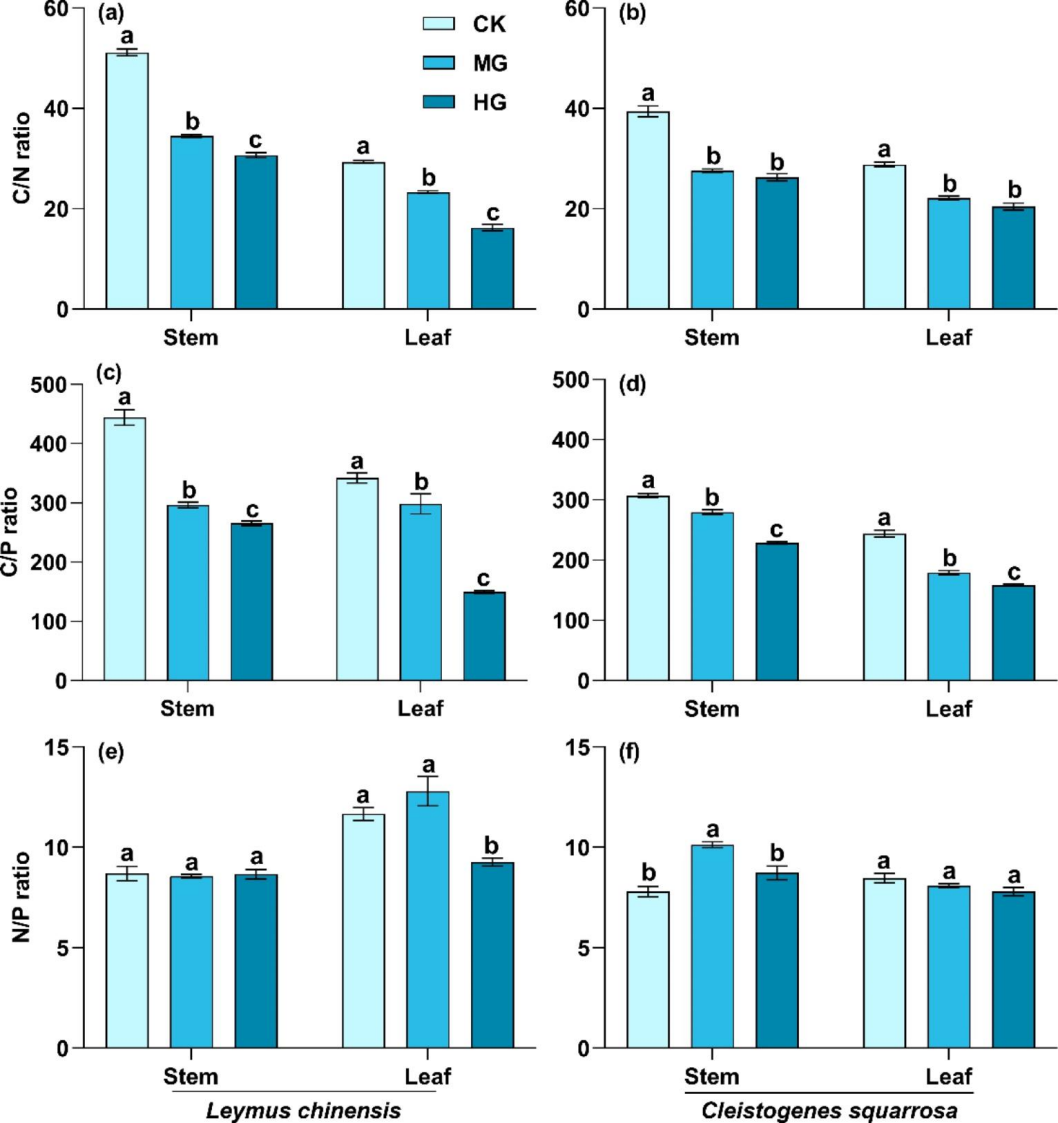
C/N, C/P, and N/P ratios in stems and leaves of *L. chinensis* and *C. squarrosa*. (a), (c), and (e): *L. chinensis;* (b), (d), and (f): *C. squarrosa.* CK: control treatment; MG: moderate grazing; HG: heavy grazing. Dif?ferent lowercase letters indicate significant differences in C/N, C/P, and N/P among the three grazing intensities at 0.05 level

C, N, and P stoichiometry of the two species had divergent responses to grazing intensity but different organs had similar responses in the grazed grasslands. C/N and C/P of stems and leaves of the two species significantly reduced with grazing intensity (Fig. 2a-d; *P* < 0.05). The C/N in stems and leaves of *C. squarrosa* showed no significant differences among the moderate grazing, heavy grazing, and control treatment. Additionally, N/P in stems and leaves of the two species showed no consistent change among the three grazing intensities (Fig. 2e-f). For example, the N/P ratio of stems in *L. chinensis* and of leaves in *C. squarrosa* had no significant response among the three grazing intensities. The N/P ratio of leaves in *L. chinensis* significantly decreased with grazing intensity.

**Fig. 3:**
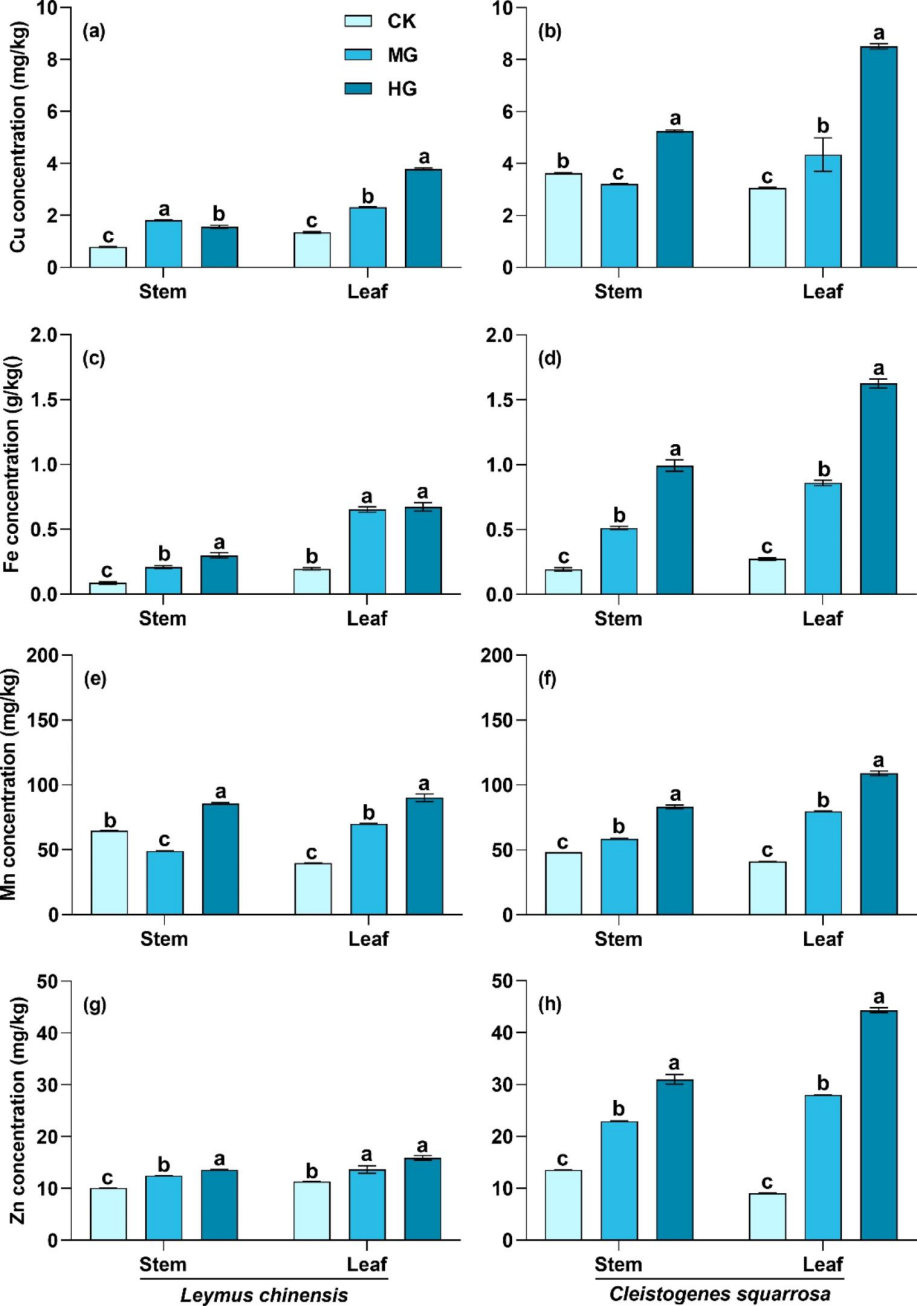
Cu, Fe, Mn, and Zn concentrations in stems and leaves of *L. chinensis* and *C. squarrosa*. (a), (c), and (e): *L. chinensis;* (b), (d), and (f): *C. squarrosa.* CK: control treatment; MG: moderate grazing; HG: heavy grazing. Different lowercase letters indicate significant differences in Cu, Fe, Mn and Zn concentrations among the three grazing intensities at 0.05 level

Microelements in stems and leaves of the two species had the same response to grazing. Generally, Cu, Fe, Mn and Zn concentrations in stems and leaves of the two species significantly increased with grazing intensity, except for Cu concentration in stems of *L. chinensis* and *C. squarrosa*, and Mn concentration in stems of *L. chinensis* (Fig. 3; *P* < 0.05). For instance, Fe concentration in stems and leaves of the two species under heavy grazing were 3.75 and 3.52 times higher than those of the control treatment, respectively.

### Nutrient reallocation in different organs and hierarchical levels under grazing

The sensitivity of microelements was higher than that of macroelements in grazed grasslands at the element level. C concentration in stems and leaves of the two species and Mn concentration in the stems of *L. chinensis* under moderate grazing had negative nutrient change rates, while other elements had positive nutrient change rates (Table 1). Generally, microelements in stems and leaves of the two species under moderate and heavy grazing had higher nutrient change rates than those in the control treatment (except for Mn and Zn concentrations in stems and leaves of *L. chinensis*), especially for *C. squarrosa* (Table 1). For example, the nutrient change rates of N and P in leaves of *C. squarrosa* under heavy grazing were only 40.4% and 55.1%, while those of Cu, Fe, Mn and Zn concentrations were much higher at 178.5%, 495.1%, 164.7% and 387.4%, respectively.

**Table 1:** Nutrient change rates in stems and leaves of *L. chinensis* and *C. squarrosa*

Elements	*L. chinensis*				*C. squarrosa*			
	**Moderate grazing**		**Heavy grazing**		**Moderate grazing**		**Heavy grazing**	
	**Stem**	**Leaf**	**Stem**	**Leaf**	**Stem**	**Leaf**	**Stem**	**Leaf**
C	-0.3	-1.8%	-0.9	-2.5%	-2.0%	-0.2%	-2.2%	-0.9%
N	28.6%	45.5%	44.1%	108.1%	39.8%	30.2%	46.6%	40.4%
P	34.8%	33.2%	54.8%	157.9%	9.7%	34.4%	33.6%	55.1%
Cu	131.1%	71.6%	99.6%	180.5%	-11.3%	42.2%	45.0%	178.5%
Fe	142.3%	232.2%	246.2%	242.4%	165.5%	214.6%	413.8%	495.1%
Mn	-24.2%	76.1%	32.1%	126.8%	22.0%	93.5%	72.7%	164.7%
Zn	23.3%	20.5%	34.5%	40.3%	69.5%	208.1%	129.1%	387.4%

**Fig. 4:**
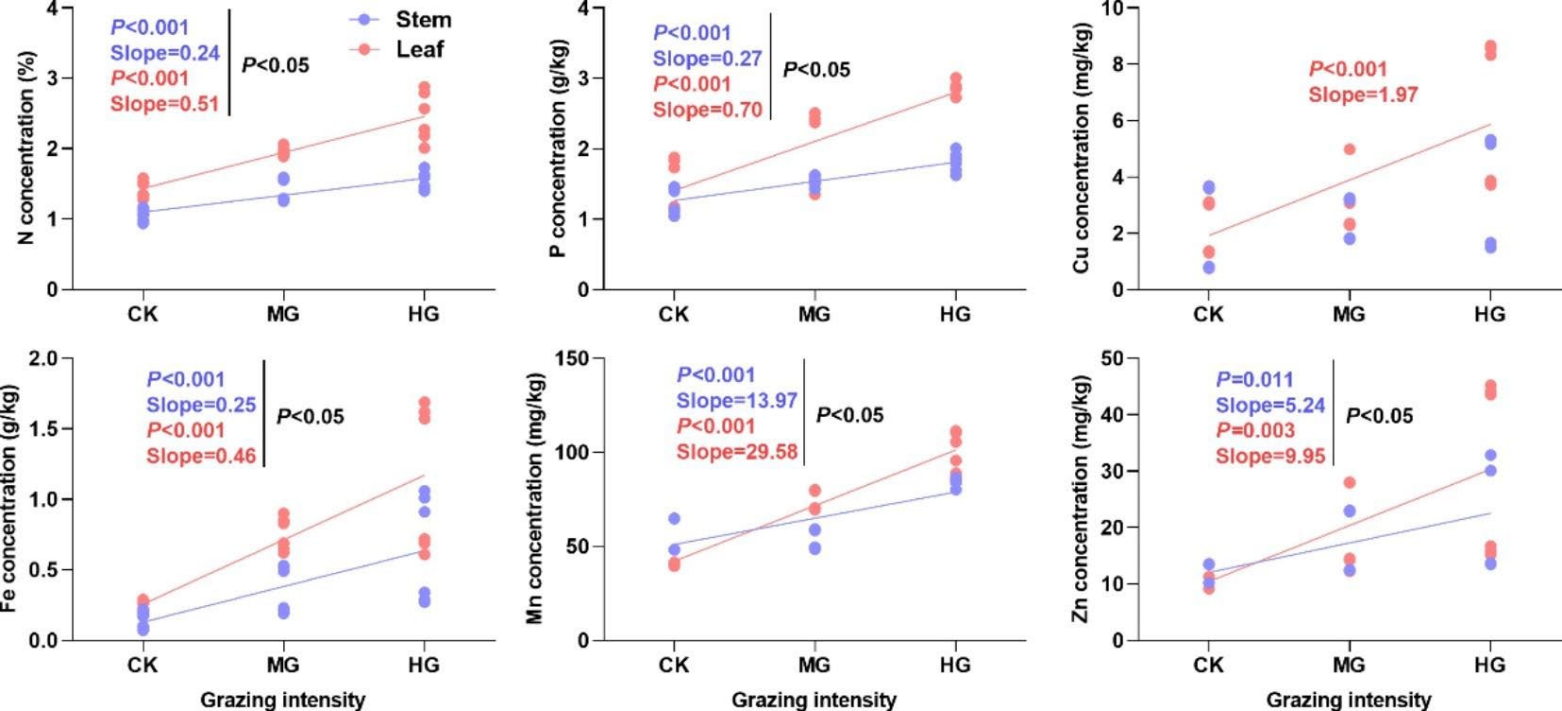
Nutrient correlations between stems and leaves with grazing intensity. CK: control treatment; MG: moderate grazing; HG: heavy grazing. Black P-value indicates significant differences in the intercept of nutrient concentrations between leaves and stems at 0.05 level

Except for Cu concentration, N, P, Fe, Mn and Zn concentrations in stems and leaves of the two species had significant positive correlations with grazing intensity (Fig. 4; *P* < 0.05). More importantly, the nutrient slopes in leaves were significantly higher than those in stems (Fig. 4; *P* < 0.05).

### Plant functional traits at individual, population and community levels under grazing

**Fig. 5:**
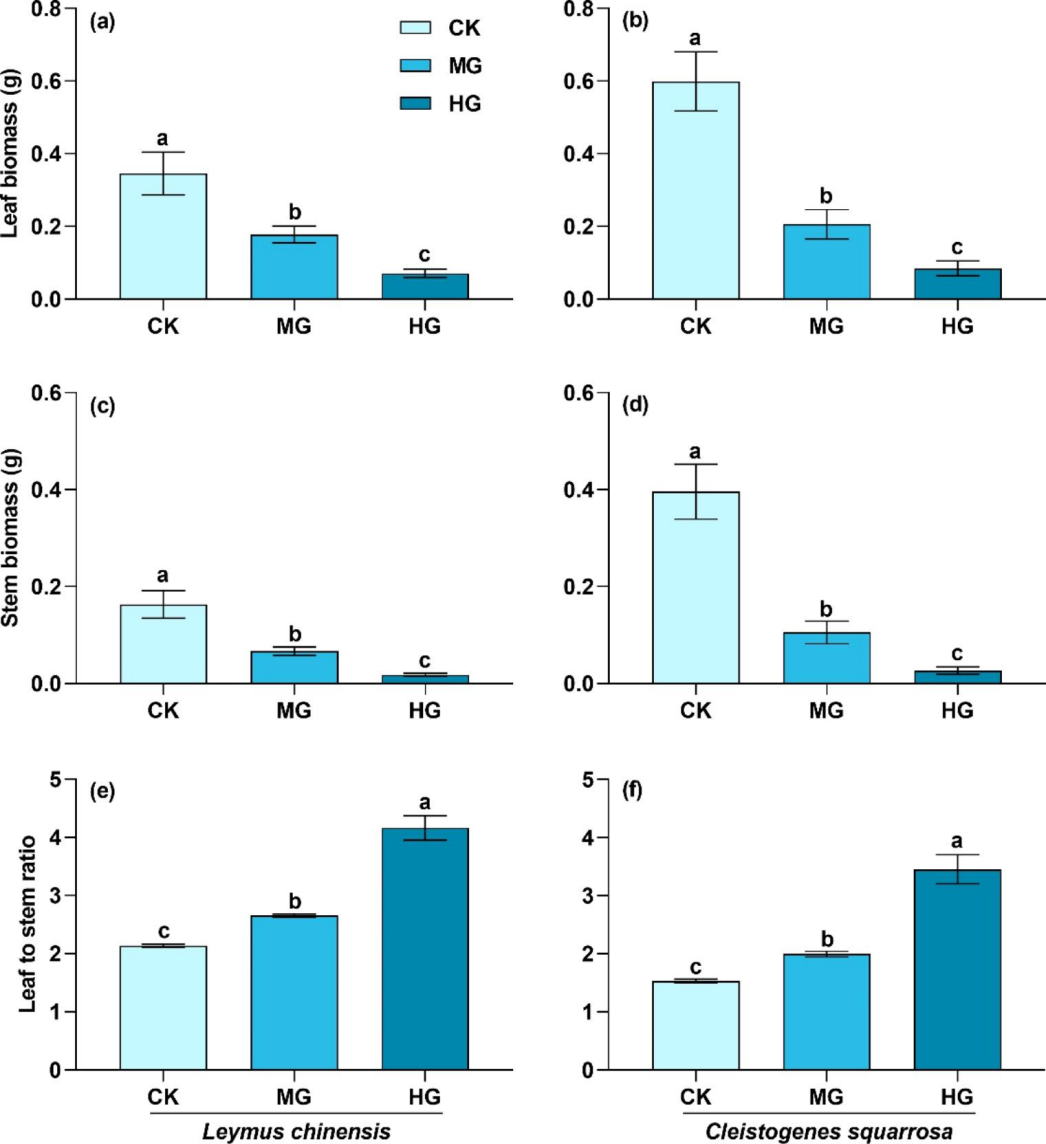
Leaf biomass, stem biomass, and leaf to stem ratio of *L. chinensis* and *C. squarrosa*. (a), (c), and (e): *L. chinensis;* (b), (d), and (f):*C. squarrosa.* CK: control treatment; MG: moderate grazing; HG: heavy graz?ing. Different lowercase letters indicate significant differences in leaf biomass, stem biomass and leaf to stem ratio among the three grazing intensities at 0.05 level

Functional traits in stems and leaves of *L. chinensis* and *C. squarrosa* had the same response to grazing at individual level. Aboveground biomass of stems and leaves in the two species significantly decreased with grazing intensity (Fig. 5a-d; *P* < 0.05). For example, aboveground biomass of stems and leaves of *L. chinensis* under heavy grazing were only 6.3% and 20.0% of those under the control treatment, while those of *C. squarrosa* were 6.7% and 14.0% of the control treatment, respectively. The biomass reallocation of stems and leaves induced by grazing altered leaf to stem ratio. The leaf to stem ratio of the two species significantly increased with grazing intensity (Fig. 5e-f; *P* < 0.05). For example, leaf to stem ratios of the two species under heavy grazing were 195.3% and 225.5% greater than those under no grazing.

**Fig. 6:**
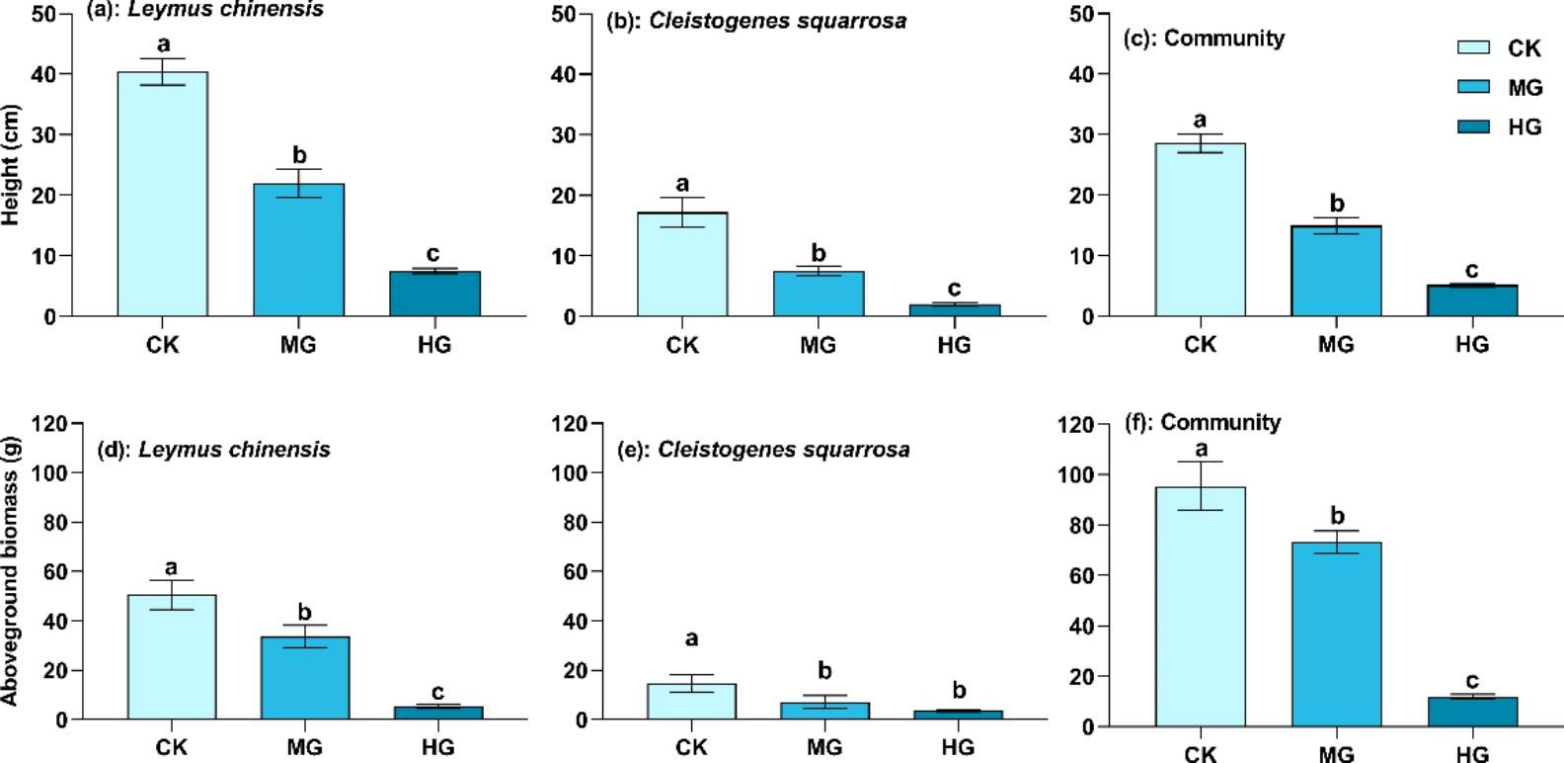
Height and aboveground biomass of *L. chinensis*, *C. squarrosa* and the plant community. CK: control treatment; MG: moderate grazing; HG: heavy grazing. Different lowercase letters indicate significant differences in height and aboveground biomass at population and community levels among the three grazing intensities at 0.05 level

Grazing had a similar effect on height and aboveground biomass at population and community levels. The height and aboveground biomass of *L. chinensis* and *C. squarrosa* at population level under moderate and heavy grazing were significantly lower than those in the control treatment (Fig. 6; *P* < 0.05). For example, under heavy grazing, the height and aboveground biomass of *L. chinensis* were 18.6% and 11.6% of those in the control treatment, while these of *C. squarrosa* were 11.9% and 19.6% of those in the control treatment. Height and aboveground biomass at the community level significantly reduced with increasing grazing intensity (Fig. 6; *P* < 0.05). The aboveground biomass of *L. chinensis* and *C. squarrosa* accounted for 59.7%~86.6% of community aboveground biomass.

### Driving factors of community structure and function changes under grazing

**Fig. 7:**
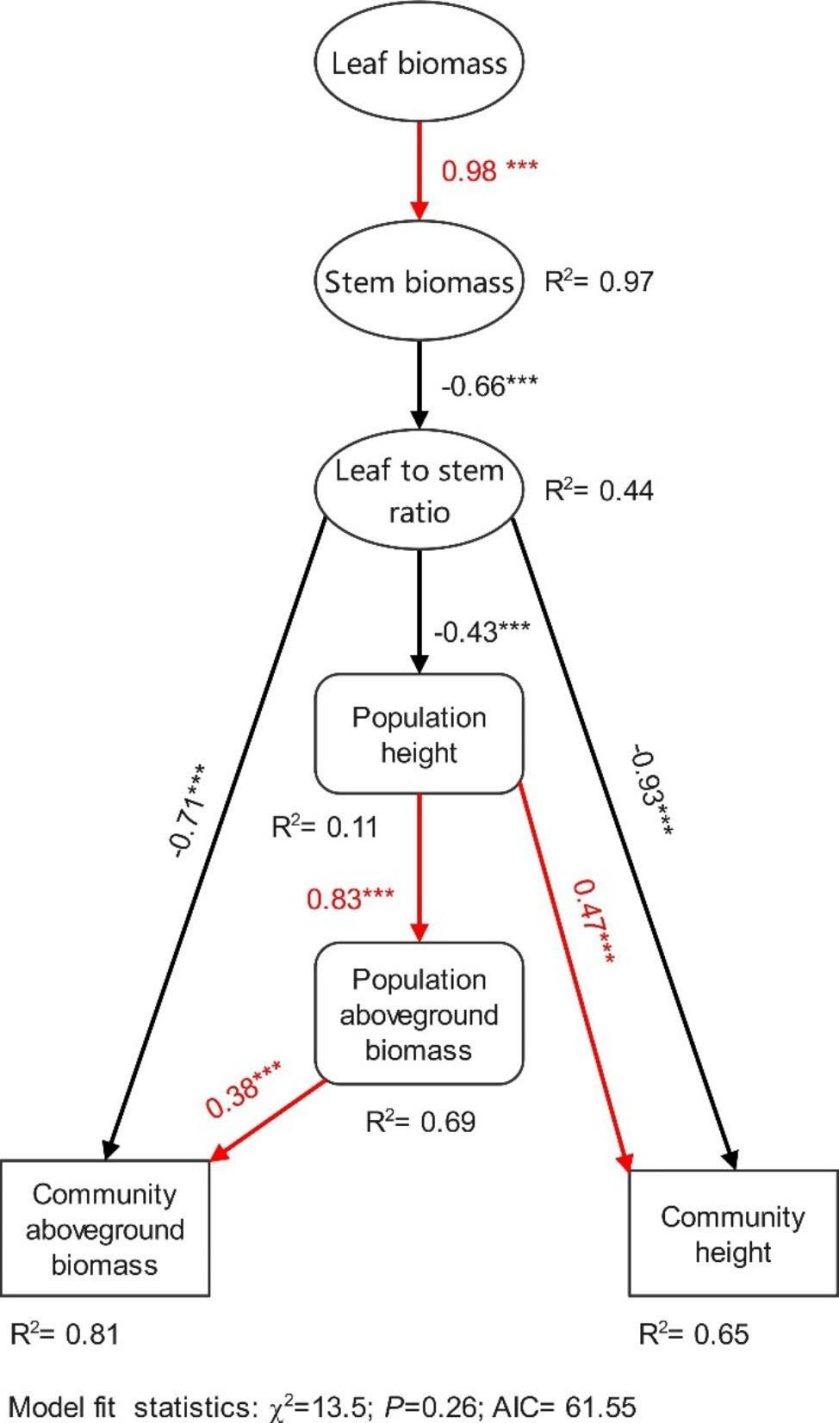
Structural equation modelling analysis of the effects of stem and leaf traits due to nutrient reallocation on population and community traits under grazing. Ellipses, oblongs and rectangles indicate measured variables at individual, population, and community levels, respectively. Red and black lines represent positive and negative correlations between the two measured variables, respectively. R^2^ value indicates the proportion of variation explained by other variables and values associated with solid arrows represent standardized path coefficients

Nutrient reallocation between stems and leaves of *L. chinensis* and *C. squarrosa* affected by grazing induced the asymmetrical allocation of aboveground biomass between these two organs, which clearly altered community structure and function in grazed grasslands. At the individual level, aboveground biomass reallocation of leaves significantly affected aboveground biomass of stems under grazing, increasing leaf to stem ratio of the two species in grazed grasslands (Fig. 7). At plant population and community levels, the increase in leaf to stem ratio directly decreased community height and aboveground biomass and indirectly reduced population height, altering the height and aboveground biomass of the plant community (Fig. 7).

## Discussion

Grazing is a main land-use practice in grassland ecosystems and directly alters nutrient cycling of the soil-plant system. Plants adapt to grazing by nutrient reallocation among different organs, altering plant community structure and functions. Plants significantly increased macroelement and microelement concentrations in stems and leaves in this grazed grassland. In the process of stem and leaf reconstruction, plants preferentially allocated more nutrient resources to leaves rather than stems, increasing leaf to stem ratio. This increase in leaf to stem ratio due to nutrient reallocation between leaves and stems directly reduced the height and aboveground biomass of the plant population and community, comprehensively driving grassland degradation in the grazed grassland.

### Effects of grazing on different nutrient elements

Grazing alters the nutrient cycling of the soil-plant system through a series of livestock behaviors (e.g., foraging, trampling, and feces and urinary return), which affects the acquisition and reallocation of nutrients in plants [26, 28]. Our study indicated that C concentration in stems and leaves of the two dominant species significantly decreased with grazing intensity (Fig. 1). This was mainly due to the damage to plant leaves by foraging and trampling of livestock, which reduced plant photosynthesis and accumulation of organic matter [16]. Previous study found that leaf C concentration significantly increased with grazing intensity, which was contrary to our study [11]. Differences in grassland type and grazing intensity may be the main reasons for these inconsistent results. Previous studies conducted in a meadow steppe in Inner Mongolia, China (i.e., with higher precipitation than typical steppe) showed that grazing benefited the compensatory growth of plants [38]. The increase in compensatory growth could reduce the negative effects of grazing on plant photosynthesis. Additionally, grazing intensity in previous studies was lower than in the present study. In our study, N and P concentrations in stems and leaves significantly increased with grazing intensity (Fig. 1), which was consistent with previous studies [32, 39]. N and P are the main components of protein and nucleic acid [19]. Plants reconstruct damaged stems and leaves by compensatory growth in grazed grasslands. These physiological processes utilize protein and nucleic acid, resulting in the increase in N and P concentrations in stems and leaves. The changes in C, N and P concentrations of stems and leaves drove the alteration in C, N, and P stoichiometry. The C/P and C/N ratios in stems and leaves significantly reduced with grazing intensity (Fig. 2), indicating that plants had higher growth rates. Compared with C/P and C/N, the N/P in stems and leaves showed no consistent changes with grazing intensity (Fig. 2). This inconsistent change in N/P showed *L. chinensis* might have a faster leaf growth rate than *C. squarrosa*. This hypothesis needs to be explored in future research. It also indicated that N and P were not the main limiting elements during grassland degradation in this region, supporting results reported by previous study [11]. However, other study found that C, N and P concentrations in leaves had no significant responses to grazing [32]. Liu et al. (2018) found that N and P concentrations in leaves significantly decreased in fenced grasslands [26]. These divergent results show that plants adapt to environmental changes with multiple complex nutrient strategies, and highlight that nutrient strategies are related to grassland management practices, disturbance duration, and the grazing tolerance of plant species.

Compared with macroelements, microelement concentrations are lower, but have important roles in regulating growth development and physiological metabolism [33]. Our study showed that Cu, Fe, Mn and Zn concentrations significantly increased with grazing intensity (Fig. 3). Microelements are the main components or electron transport receptors of enzymes in photosynthesis, respiration, N and P metabolism, and other physiological activities in plants [34, 35]. For example, Fe is an important electron transport receptor in plant respiration, and Zn and Mn are related to P metabolism in plants. The alteration in photosynthetic rate and respiration rate due to compensatory growth of plants induced the increase in microelement concentrations in grazed grasslands [40]. Moreover, some microelements are involved in N and P metabolism in plants [33, 40]. With the synthesis of protein and nucleic acid in the process of stem and leaf reconstruction, microelements also significantly increase in grazed grasslands. Compared with macroelements, microelements in stems and leaves had higher nutrient change rates (Table 1). This might be because microelements had a more sensitive response to grazing [29]. On the other hand, it may be due to their relatively low concentration in plants. These two speculations need to be verified in future studies. There were some differences in Mn concentrations in stems of *L. chinensis* and *C. squarrosa*. This might be due to random variation among the plant samples. The changes in macroelement and microelement concentrations in stems and leaves due to grazing mean that plants adapted to grazing by regulating nutrient concentration rapidly in grazed grasslands, which further had intensive effects on plant functional traits, and quantitative characteristics of the population and even the plant community.

### Effects of grazing on nutrient reallocation in stems and leaves

Although grazing significantly increased plant nutrient concentrations, nutrient concentrations in stems and leaves had divergent responses to grazing. This study found that the nutrient slopes of macroelements and microelements in leaves were higher than those in stems (Fig. 4). This result indicated that plants altered nutrient allocation strategy and preferentially allocated nutrient resources to leaves. Plants might have faster growth rates after this regulation, especially under heavy grazing. Previous studies showed that plants regulated nutrient resource strategy when nutrients were limited and preferentially allocated nutrient resources to assimilation organs [28]. Livestock behaviors affect photosynthesis, respiration and other physiological activity of plants [16]. Foraging and trampling by livestock directly damaged plant stems and leaves, which altered the functional traits of stems, leaves, and even plant individuals [41]. Moreover, other livestock behaviors directly or indirectly affect soil physicochemical properties (e.g., soil temperature, soil moisture, and soil nutrient), which decrease the absorption of water and nutrients [17, 42]. These ecological processes might reduce photosynthetic rate, respiration rate, water use efficiency, and nutrient use efficiency of plants, which decreases photosynthate accumulation and result in C deficit of plants [16, 43]. Leaves are the main organ involved in photosynthesis, transpiration, and nutrient utilization. Thus, plants preferentially allocated nutrient resources to leaves to acquire maximum photosynthetic rate during leaf reconstruction under conditions of limited access to resources in grazed grasslands [44]. Additionally, Williamson et al. (2012) found that decline in light stimulated competition of plants for light and preferentially allocated nutrient resources to stems, which increased the growth rate of stems [45]. The decrease in community cover and increase in light due to livestock foraging reduced the competition of plants for light [46]. This ecological process might drive nutrient allocation to leaves rather than stems. Regretfully, these two speculations about the asymmetrical allocation of nutrients between stems and leaves under grazing could not be verified in this study and need to be further studied in the future. Liu et al. (2018) found that nutrient resources were preferentially allocated to plant stems in fenced grasslands in Inner Mongolia [26]. Grazing and fencing drive retrogressive and restoration successions of grassland ecosystems, respectively, leading to divergent patterns of nutrient allocation by plants. These different results also demonstrated that the nutrient allocation between stems and leaves had compensatory effects, which may be determined by environmental changes. The nutrient strategy of asymmetric allocation between stems and leaves under grazing is of great significance for life history and survival strategy of plants [24, 28]. In grazed grasslands, plants allocate more nutrient resources to leaves, which not only optimally uses limited resources, but also improves resource utilization efficiency and enables plants to better adapt to grazing disturbance [44]. The regulation of nutrient strategy might depend on grazing regime (e.g., continual grazing and rotation grazing) or grassland type (e.g., steppe, prairie, and savanna). Moreover, this phenomenon signals a shift from growth strategy to defense strategy and indicates a variety of survival strategies for plants in different environments.

### Nutrient reallocation between stems and leaves alters community structure and function

Plant functional traits determine the characteristics of the population and community, and are also the key to assessing the degradation of grassland ecosystem productivity in grazed grasslands [47], and their study has become a hotspot in grazed grassland research. Nutrient reallocation between stems and leaves has significant effects on plant functional traits in grazed grasslands [28, 44]. Our study found that aboveground biomass of stems and leaves significantly reduced but leaf to stem ratio increased with grazing intensity (Fig. 5), indicating that leaf reconstruction was preferred over stems under grazing from the perspective of plant functional traits. The nutrient trade-off strategy between stems and leaves was the main factor driving the changes in stem and leaf aboveground biomass under grazing [44]. The increase in leaf to stem ratio under grazing meant that stem traits were more sensitive than leaf traits. Additionally, previous studies found that stem traits (e.g., stem length, stem diameter, and internode number) were more sensitive than leaf traits (e.g., leaf thickness and leaf number) in fenced grasslands in Inner Mongolia [26]. These studies showed that plants might regulate stem traits to adapt to environmental changes in grassland ecosystems. Compared with independent functional traits, such as stem and leaf traits, leaf to stem ratio can be regarded as a comprehensive index to evaluate the responses of allocation strategies of stems and leaves to environment changes. It is also more sensitive to grassland management measures (e.g., grazing and mowing) and could be used as a monitoring index in grassland management in the future. Moreover, the shortened stem due to reduced nutrient allocation to stems also decreased the foraging of livestock on plant individuals and improved the adaptability of plants to grazing disturbance in grazed grasslands [11].

Change in plant functional traits could alter the structure, function, and diversity of the plant community [9]. This study found that height and aboveground biomass at the population and community levels significantly decreased with grazing intensity (Fig. 6). This phenomenon is widespread in grasslands worldwide and has been a focus of much research [48–50]. Some studies have revealed the driving mechanisms of grassland community structure and function degradation due to grazing from the perspectives of interspecific relationships, soil-plant nutrient cycling, and microenvironmental change [7, 17, 51]. Other researchers have proposed the “grazing avoidance hypothesis” and “growth inhibition hypothesis” to explain this phenomenon [11]. In this study, the asymmetric nutrient allocation between stems and leaves drove the redistribution of plant stem and leaf aboveground biomass, which further led to the decrease in height and aboveground biomass at population and community levels (Fig. 7). These ecological processes comprehensively induced grassland community degradation, indicating that the nutrient allocation strategies of plants had intensive effects on the grassland ecosystem. Our study also supported the findings of He et al. (2021) from a nutrient reallocation perspective [47]. Moreover, this research proposed an underlying mechanism of grassland ecosystem structure and function degradation driven by grazing at the individual, population, and community levels.

## Conclusion

Different plants organs intensely compete for nutrient resources in grassland ecosystems. Grazing enhances nutrient reallocation among different organs by altering plant nutrient acquirement, resulting in the regulation of tradeoff strategies between growth and defense. Grazing significantly increased macroelement and microelement concentrations in stems and leaves and induced preferential allocation of nutrients to assimilation organs. Grazing asymmetrically decreased the aboveground biomass of stems and leaves, increasing leaf to stem ratio. The increase in leaf to stem ratio due to changes in nutrient reallocation in stems and leaves significantly reduced height and aboveground biomass at population and community levels, resulting in grassland ecosystem degradation. Our study revealed the underlying mechanism of grassland ecosystem degradation induced by grazing from the perspective of nutrient resource reallocation.

## Data Availability

The data are available from the corresponding author.
